# Methodological Considerations in Estimation of Phenotype Heritability Using Genome-Wide SNP Data, Illustrated by an Analysis of the Heritability of Height in a Large Sample of African Ancestry Adults

**DOI:** 10.1371/journal.pone.0131106

**Published:** 2015-06-30

**Authors:** Fang Chen, Jing He, Jianqi Zhang, Gary K. Chen, Venetta Thomas, Christine B. Ambrosone, Elisa V. Bandera, Sonja I. Berndt, Leslie Bernstein, William J. Blot, Qiuyin Cai, John Carpten, Graham Casey, Stephen J. Chanock, Iona Cheng, Lisa Chu, Sandra L. Deming, W. Ryan Driver, Phyllis Goodman, Richard B. Hayes, Anselm J. M. Hennis, Ann W. Hsing, Jennifer J. Hu, Sue A. Ingles, Esther M. John, Rick A. Kittles, Suzanne Kolb, M. Cristina Leske, Robert C. Millikan, Kristine R. Monroe, Adam Murphy, Barbara Nemesure, Christine Neslund-Dudas, Sarah Nyante, Elaine A Ostrander, Michael F. Press, Jorge L. Rodriguez-Gil, Ben A. Rybicki, Fredrick Schumacher, Janet L. Stanford, Lisa B. Signorello, Sara S. Strom, Victoria Stevens, David Van Den Berg, Zhaoming Wang, John S. Witte, Suh-Yuh Wu, Yuko Yamamura, Wei Zheng, Regina G. Ziegler, Alexander H. Stram, Laurence N. Kolonel, Loïc Le Marchand, Brian E. Henderson, Christopher A. Haiman, Daniel O. Stram

**Affiliations:** 1 Department of Preventive Medicine, Keck School of Medicine and Norris Comprehensive Cancer Center, University of Southern California, Los Angeles, CA, United States of America; 2 Sylvester Comprehensive Cancer Center and Department of Epidemiology and Public Health, University of Miami Miller School of Medicine, Miami, FL, United States of America; 3 Department of Cancer Prevention and Control, Roswell Park Cancer Institute, Buffalo, NY, United States of America; 4 The Cancer Institute of New Jersey, New Brunswick, NJ, United States of America; 5 Division of Cancer Epidemiology and Genetics, National Cancer Institute, National Institutes of Health, Bethesda, MD, United States of America; 6 Division of Cancer Etiology, Department of Population Science, Beckman Research Institute, City of Hope, CA, United States of America; 7 International Epidemiology Institute, Rockville, MD, United States of America; 8 Division of Epidemiology, Department of Medicine, Vanderbilt Epidemiology Center, Vanderbilt University and the Vanderbilt-Ingram Cancer Center, Nashville, TN, United States of America; 9 The Translational Genomics Research Institute, Phoenix, AZ, United States of America; 10 Epidemiology Program, Cancer Research Center, University of Hawaii, Honolulu, HI, United States of America; 11 Cancer Prevention Institute of California, Fremont, CA, United States of America; 12 Epidemiology Research Program, American Cancer Society, Atlanta, GA, United States of America; 13 Division of Public Health Sciences, Fred Hutchinson Cancer Research Center, Seattle, WA, United States of America; 14 Division of Epidemiology, Department of Environmental Medicine, New York University Langone Medical Center, New York, NY, United States of America; 15 Chronic Disease Research Centre and Faculty of Medical Sciences, University of the West Indies, Bridgetown, Barbados; 16 Department of Preventive Medicine, Stony Brook University, Stony Brook, NY, United States of America; 17 Division of Epidemiology, Department of Health Research & Policy, Stanford University School of Medicine and Stanford Cancer Institute, Stanford, CA, United States of America; 18 Department of Medicine, University of Illinois at Chicago, Chicago, IL, United States of America; 19 Department of Epidemiology, Gillings School of Global Public Health, and Lineberger Comprehensive Cancer Center, University of North Carolina, Chapel Hill, NC, United States of America; 20 Department of Urology, Northwestern University, Chicago, IL, United States of America; 21 Department of Biostatistics and Research Epidemiology, Henry Ford Hospital, Detroit, MI, United States of America; 22 Cancer Genetics Branch, National Human Genome Research Institute, National Institutes of Health, Bethesda, MD, United States of America; 23 Department of Pathology, Keck School of Medicine and Norris Comprehensive Cancer Center, University of Southern California, Los Angeles, CA, United States of America; 24 Department of Epidemiology, Harvard School of Public Health, Boston, MA, United States of America; 25 Department of Epidemiology, The University of Texas M.D. Anderson Cancer Center, Houston, TX, United States of America; 26 Institute for Human Genetics, Department of Epidemiology and Biostatistics, University of California San Francisco, San Francisco, CA, United States of America; The University of Texas Health Science Center (UTHSCSA), UNITED STATES

## Abstract

Height has an extremely polygenic pattern of inheritance. Genome-wide association studies (GWAS) have revealed hundreds of common variants that are associated with human height at genome-wide levels of significance. However, only a small fraction of phenotypic variation can be explained by the aggregate of these common variants. In a large study of African-American men and women (n = 14,419), we genotyped and analyzed 966,578 autosomal SNPs across the entire genome using a linear mixed model variance components approach implemented in the program GCTA (Yang et al Nat Genet 2010), and estimated an additive heritability of 44.7% (se: 3.7%) for this phenotype in a sample of evidently unrelated individuals. While this estimated value is similar to that given by Yang et al in their analyses, we remain concerned about two related issues: (1) whether in the complete absence of hidden relatedness, variance components methods have adequate power to estimate heritability when a very large number of SNPs are used in the analysis; and (2) whether estimation of heritability may be biased, in real studies, by low levels of residual hidden relatedness. We addressed the first question in a semi-analytic fashion by directly simulating the distribution of the score statistic for a test of zero heritability with and without low levels of relatedness. The second question was addressed by a very careful comparison of the behavior of estimated heritability for both observed (self-reported) height and simulated phenotypes compared to imputation *R^2^* as a function of the number of SNPs used in the analysis. These simulations help to address the important question about whether today's GWAS SNPs will remain useful for imputing causal variants that are discovered using very large sample sizes in future studies of height, or whether the causal variants themselves will need to be genotyped de novo in order to build a prediction model that ultimately captures a large fraction of the variability of height, and by implication other complex phenotypes. Our overall conclusions are that when study sizes are quite large (5,000 or so) the additive heritability estimate for height is not apparently biased upwards using the linear mixed model; however there is evidence in our simulation that a very large number of causal variants (many thousands) each with very small effect on phenotypic variance will need to be discovered to fill the gap between the heritability explained by known versus unknown causal variants. We conclude that today's GWAS data will remain useful in the future for causal variant prediction, but that finding the causal variants that need to be predicted may be extremely laborious.

## Introduction

Hundreds of genome-wide association studies (GWAS) since 2005 have reported over 5,000 common genetic variants that were found to be associated with over 200 diseases and traits (http://www.genome.gov/gwastudies/) [1]. Nevertheless the proportion of phenotype variance or disease risk that common variants can possibly contribute to is controversial. For example, two research groups, Lango Allen et al (2010) and Yang et al (2010) estimated the heritability of height that is explained by common variants and drew somewhat different conclusions [2, 3]. We investigated several issues regarding the interpretation of height heritability using the linear mixed model approach described by Yang et al [3]. These issues are

1. The degree to which estimates of heritability obtained using the LMM approach are dependent upon the existence of subtle population structure (i.e. hidden relatedness) in order to ensure that variance components are identifiable (see below).
2. As described in [4] we are also interested in whether or not the additive heritability of height estimated in the linear mixed model method (LMM) applied to GWAS data can be attributed to
  - dissection of distant relatedness encompassing the complete additive phenotypic effects of all potential variants whether measured or unmeasured or
  - the additive phenotypic effects of only those SNPs that are measured in GWAS studies plus the variants in high linkage disequilibrium (LD) with these SNPs (i.e. which are highly imputable using these SNPs.)

Several authors have raised concerns about the behavior of the LMM applied to GWAS data in the presence of population structure either due to gross population stratification or more subtle relatedness [5, 6]. We undertook a more thorough analysis of the issue of the effect of small amounts of residual relatedness in GWAS data on heritability estimation using the LMM than has been described before.

If (2b) holds then this implies that currently used GWAS data will retain their value far longer than if only (2a) is true, i.e. while identifying which variants are causal may require very large sample sizes and detailed functional analysis in future work, once the causal variants are identified they may be imputed using today's existing genotyping technology. Here we say that the measured SNPs are good surrogates for the causal variants and a good predictor variable (highly correlated with height in unrelated samples) can be made up from the surrogates. On the other hand, if (2a) is driving the results of the LMM, then it will likely be necessary to greatly expand the number of variants (or surrogates) that need to be directly genotyped in order to capture the estimated height heritability in a prediction equation made up of the genotyped SNPs.

In order to address this question of the effect of low levels of hidden relatedness we undertake an extensive analysis of imputation behavior within a large GWAS. We assembled a large sample of men and women of African ancestry from 21 studies in order to explore how much of the heritable component of height is attributable to common variants in this population, and we conduct analyses of the observed height data and of simulated phenotype data (keeping the genotypes and simulating a continuous phenotype in place of observed height according to known polygenic models).

A related question concerns the expected performance of the method in the absence of population stratification and relatedness. Our concern is that the model for the variance of the outcome (i.e. height) may become unidentifiable if enough independent SNPs are used in the analysis. Here we are taking note of the fact that the genetic similarity matrix **G** used by Yang et al can be expected to approach (as the number of SNPs increase) an estimate of twice the kinship matrix and this is equal to the identity matrix if there is no population stratification or relatedness. As described below, the variance components model used to estimate heritability is unidentifiable if **G** is exactly equal to the identity matrix. Therefore it is possible that GWAS that rely upon many hundreds of thousands of markers may require some level of hidden structure or relatedness to estimate heritability precisely. We studied the behavior of the estimate of heritability as sample size increases and as the number of independent SNP predictors increases.

## Methods

### Statistical Models

There are a few different approaches used in polygenic analysis. Commonly used approaches include the omnibus (global) analysis, the score statistics approach, and kernel machine methods [2, 7–11]. The variance components approach used in Yang et al is one special case of kernel machine methods, which projects the similarity in height on a genetic similarity matrix (**G**) equivalent to the Balding-Nichols matrix [3, 12].

The method features a mixed model

y=μ+Xα+Zu+ε (1)

where **α** is a vector of fixed effects, **Z** is a genotype matrix of common variants, and is column-standardized to zero mean and unit variance. The risk coefficients associated with these variants are treated as random effects with effect size **u**, **u** ~ N(**0**, σ_u_^2^ **I**). The residual terms are distributed as multivariate normal N(**0**, σ_e_^2^ **I**). The polygene term **Zu**, with the j^th^ element equal to $\sum_{i=1}^{M}z_{ij}u_{i}$, where M is the number of markers in the model, has variance σ_g_^2^ = Mσ_u_^2^ as genotypes are standardized and assumed to be independent. Therefore we have

$$Var\left(y\right)\;=\;\sigma_{u}{}^{2}ZZ’\;+\;\sigma_{e}{}^{2}I=\;\sigma_{g}{}^{2}G+\;\sigma_{e}{}^{2}I$$ (2)

where **G** = **ZZ**’/M, or

$$G_{ij}=\frac{1}{M}\sum_{k=1}^{M}\frac{\left(z_{ik}-2f_{k})(z_{jk}-2f_{k}\right)}{2f_{k}(1-f_{k})}$$

and *f*_*k*_ is the allele frequency of the k^th^ marker. Here M is generally very large, i.e. corresponding to the hundreds of thousands of SNPs on a typical GWAS array Note that the **G** matrix is 2 times the Balding-Nichols matrix which is used as an estimator of kinship coefficients between individuals based upon SNP data [12].

The **G** matrix is commonly used for correcting association studies for population stratification, admixture and/or relatedness [12], such as in the principal components analysis (PCA) described by Price et al (2006) [13] and the Efficient Mixed-Model Association eXpedited (EMMAX) method described by Kang et al (2010) [14], both of which are widely employed by researchers to control for hidden population structure and relatedness. Because of the identity of the methods used by Yang et al to estimate height heritability (which they attribute to the specific SNPs genotyped plus variants for which the SNPs are good surrogates) and the method of Kang et al to control for hidden structure and relatedness, it is natural to wonder whether weak hidden relatedness still evident in nominally unrelated people may be influencing the heritability estimates. More precisely, if there are only very close relatives in the sample then we would expect that a relatively small number of SNPs would be able to estimate kinship between the relatives and hence provide an estimate of additive heritability by relating similarity in phenotype to similarity in kinship. It would be wrong to think, however, that any predictor equation made up of only those SNPs could be expected to provide good estimation of the phenotype of interest in another sample, whether the new sample consists of (a different set of) close relatives, or of unrelated individuals. That is, we would not want to misattribute the additive heritability estimate based on kinship to the effects of the measured SNPs themselves. A primary concern in this paper is whether this kind of misattribution of effect may be happening when nominally unrelated individuals are used, given that no set of individuals can possibly be truly unrelated.

As a motivation of these concerns consider the model for multivariate normal outcome *Y* with mean and variance defined as

$$\begin{array}{l} E\left(Y\right)\;=X\beta \\ \text{and}\\ Var\left(Y\right)\;=\;\sigma_{g}{}^{2}G+\sigma_{e}{}^{2}I \end{array}$$ (3)

From Anderson [15] it can readily be shown that the score statistic for testing for nonzero $\sigma_{g}^{2}$ in (3) can be written as

$$T^{2}=N{\frac{\left[\frac{\left(Y-X\hat{\beta })\prime G(Y-X\hat{\beta }\right)}{{\hat{\sigma }}^{2}}-\text{tr}(G)\right]}{N\text{tr}(GG)-\text{tr}{\left(G\right)}^{2}}}^{2}$$ (4)

Where *N* is sample size and $\hat{\beta }$ and *σ* ^2^ are the maximum likelihood estimates of the slope and variance parameters for fitting model (3) under the null model with $\sigma_{g}^{2}=0$. Notice that if the genetic matrix **G** is equal to the identity matrix then this quantity is 0/0, or undefined. Under an assumption that all individuals are unrelated it may be expected that as the number of SNPs increases, the estimate of **G**, namely $\frac{1}{M}ZZ\prime$, will tend towards the identity matrix **I** (since all columns of matrix **Z** have mean zero, variance 1, and the different elements are independent). If the score test is undefined with **G** = **I** then we expect that there will be loss of power to detect a non-zero $\sigma_{g}^{2}$ as **G** → **I**. On the other hand, if **G** is bounded away from **I**, this should avoid the power problem although it may complicate the interpretation of the results of the test. This suggests to us that some population stratification or relatedness may actually be needed in order to achieve well-behaved estimates, at least in certain circumstances.

Our first simulation is based on this score test statistic. For N individuals each with M SNPs we generate outcomes *Y* from model (3) by randomly sampling the columns, Z_j_ for j = 1,…, M, of **Z** (the normalized SNPs) either from a distribution with mean E(*Z*) = 0 and Var(*Z*) = **I** or from a distribution with mean E(*Z*) = 0 and Var(*Z*) = **K**, with **K** having ones on the diagonal and off diagonal elements uniformly distributed between 0 and 0.05, corresponding to a modest amount of hidden relatedness. Finally outcome Y is generated as multivariate normal with mean 0 and Var(*Y*) = σ_g_^2^ **G** + σ_e_^2^ **I**, with **G** = $\frac{1}{M}ZZ\prime$. In the simulations we keep σ_g_^2^ = σ_e_^2^ so that true underlying heritability is set to ½. This simulation approach allows us to directly investigate the distribution of *T* ^*2*^ in (4) as a function of N, M, and Var(**Z**), as well as other related issues discussed below.

In addition to the analysis of purely simulated data we further investigate our primary concerns by conducting the simulation experiment described below using both real and simulated phenotypes and/or genotypes, with the source of actual phenotype and genotype data being a combination of two large GWAS studies, conducted among men and women of African ancestry. There are some minor complications in our use of African ancestry samples in these experiments, due to recent admixture, since pairs of individuals with higher non-African ancestry (e.g. European) will have kinship coefficients that are exaggerated by the similarity in ancestry, and are not interpretable as estimates of identity by descent (IBD) sharing [16]. We minimize this aspect of the data in several ways, first by removing individuals who are the most “similar” to other individuals from our primary dataset (see below) and second by always including leading principal components as adjustment variables in our statistical analyses when estimating heritability for either observed or simulated outcomes. Moreover we note that non-African admixture proportion (as captured by leading eigenvectors) was not significantly related to height in our data.

Our simulation experiment was as follows

1. We estimated relatedness using the full set of SNP data and removed individuals who were related to one or more others with relationship coefficient (equal to twice the kinship coefficient) > 0.025
2. We estimated height heritability using the self-reported height data available from the GWAS study using all SNPs (nearly 1 million were measured).
3. We randomly divided the combined study into two sets of equal size, we labeled one set the "reference panel" and the second set the "main study"
4. Using a pruned set of SNPs (about 453,000 SNPs chosen as described below) we then masked 1,000 SNPs so that they were available in the reference panel but not in the main study.
5. We then simulated phenotype data using the 1,000 SNPs as causal SNPs with heritability set to 50% of phenotype variance, for both reference panel and main study participants
6. Using the pruned SNPs we phased both reference panel and the main study data using SHAPEIT [17]
7. Using the haplotypes in the reference panel we then
  - Imputed the 1,000 masked causal SNPs in the main study using IMPUTE2 [18] using the non-masked (measured) data. We computed an average imputation *R* ^*2*^ by comparing the imputations for the masked SNPs with the true genotypes for the same SNPs in the main study
  - In the main study we estimated heritability of true height using the measured SNPs using the LMM described above
  - In the main study we also estimated heritability of simulated phenotypes using the measured SNPs

We then randomly removed from consideration half the measured SNPs and repeated step 7, and then repeated step 7 again after removing 3/4 of the measured SNPs and finally once again after removing 7/8 of the measured SNPs. In each case we are interested in how the heritability estimates and the imputation *R* ^*2*^ estimates varied with the number of measured SNPs. More precisely we expected that heritability will decline approximately with the average imputation *R* ^*2*^. If, as the number of SNPs used in the analyses and simulations is reduced, the heritability estimates using either the simulated or true phenotypes remained higher than expected given the corresponding imputation *R* ^*2*^, then we may interpret the results of the heritability estimation in the real data as reflecting residual relatedness and therefore a misattribution of effect from unmeasured to measured SNPs.

In addition to seeing how well the various subsets of SNPs predicted the unmeasured SNPs in the reference panel we also estimated the ability of each subset to impute the common (MAF > 1%) SNPs in the 1,000 Genomes Study. We used the combination of SHAPEIT and IMPUTE2 to impute these SNP panels for all unrelated individuals described above with the March 2012 1,000 Genomes Study serving as a cosmopolitan reference panel.

### Ethics Statement

The Institutional Review Board at the University of Southern California approved the study protocol.

### Study Population

This study includes women and men of African ancestry from 9 epidemiological studies of breast cancer and 12 epidemiological studies of prostate cancer, which comprise a total sample size of 15,032 (5,984 women and 9,048 men). Please refer to S1 File for a brief description of each of the studies.

### Genotyping and Quality Control

Genotyping was conducted on Illumina 1M-Duo BeadChip. Of 15,032 DNA samples available we excluded 613 with either low DNA concentrations, unexpected sex chromosome heterozygosity (i.e. conflicting or ambiguous sex based on the X chromosome genotypes when compared to reported sex), call rates < 95%, or < 5% African ancestry. The resulting dataset included genotypes for a total of 14,419 participants. Starting with 1,153,397 SNPs, we removed SNPs on sex chromosomes (n = 34,504), with <95% call rate, MAFs <1%, or P value for Hardy-Weinberg equilibrium (HWE) <1×10^−7^(n = 142,339). Randomly selected SNPs that are not located within the known risk loci of height were used to infer the principal components (PCs) (n = 9,976) and excluded from analysis. (We have previously found that including even 2,500 randomly selected SNPs in PC analysis, is more than sufficient to correct for admixture and other gross stratification in a study of multiethnic samples, including African American, see [19]). The final analysis included 966,578 autosomal SNPs among 14,419 subjects.

### Analysis of African Ancestry GWAS

In the analysis of the observed genotypes and either simulated or observed phenotypes for the African ancestry GWAS we used the principal components approach to control for the effects of admixture or other gross population substructure in analyses of both real and simulated data. We used 9,976 SNPs randomly selected from the genome, excluding known risk loci for height to infer PCs. Known risk loci for height were identified from a catalog of published GWAS (http://www.genome.gov/gwastudies/) listed on the webpage of the National Human Genome Research Institute, including GWAS scan results from Lango Allen et al (2010), and a GWAS scan in individuals of African ancestry by N’Daiye et al (2011) [2, 20]. We included the first 10 PCs in all models to control population structure.

### Further Simulation Details

#### Distribution of T^2^

For the first set of simulations we directly simulated the score test statistic. Here we report the mean value of T^2^ and its standard error over the simulations, as well as the fraction of simulations for which T^2^ was greater than an empirically determined 95 percent critical value, obtained by simulation under the null hypothesis. We performed this additional null simulation because, while typically score tests such as used here are asymptotically distributed as a chi-square random variable with 1 degree of freedom, the asymptotics may not apply in the case when the genetic matrix **G** approaches the identity matrix because of the lack of model identifiability described above.

#### Estimating heritability explained by common variants

We employed a variance components approach above to estimate the heritability explained by common SNPs from an additive polygenic model [3].

We fit several LMMs using the Genome-wide Complex Trait Analysis (GCTA) tool. Fixed effects included age, sex, study site and the first 10 PCs.

#### Estimating additive and epistatic components of variance

We used the—genome command in PLINK [21] (http://pngu.mgh.harvard.edu/purcell/plink/) to estimate the probability of pair-wise IBD (Pr(IBD = 0), or z0; Pr(IBD = 1), or z1; Pr(IBD = 2), or z2) for all pairs in the sample using genotype information from all the SNPs. Following that we constructed an “unrelated” (only distantly related) sample by dropping one of each pair of related individuals (relationship coefficient k = 1/2 z1 + z2 > 0.025). This left 12,488 individuals remaining. On the other hand, by considering higher IBD sharing pairs, we constructed two related samples (z1> = 0.3, n = 1,415 and z1> = 0.4, n = 575) and again estimated heritability of height using the variance components approach. We argue that the resulting estimate may include both additive and epistatic components as well as the effects of shared environment, the latter two components are assumed to be less important for distant than for close relationships, and to die off more quickly than the additive component *h* ^*2*^ as relationships become more distant. Of course the estimate using closely-related individuals will also reflect the effects of any variants (such as rare causal SNPs) that are not in strong LD with the GWAS SNPs but which nevertheless are more similar within sets of close relatives than between them.

#### Pruning of SNPs

We pruned SNPs from the whole genome and kept only those with multiple-tagging correlation coefficient (*R* ^*2*^) less than 0.25 using PLINK [21] using a 50 SNP window (http://pngu.mgh.harvard.edu/purcell/plink/). Following that, we randomly selected 1,000 SNPs from the pruned SNP set and treated them as causal SNPs.

#### Simulated Phenotypes

A random effects model was assumed where the effect size u was sampled from N(0,1). Upon calculating the polygenic genetic score g, a residual effect e was sampled from N(0, σ_g_^2^ (1-*h* ^*2*^)/*h* ^*2*^) to set the heritability of the phenotype to *h* ^*2*^. Phenotype data were then generated by summing the additive genetic effects and residual effects.

## Results

### Distribution of T^2^

Table 1 shows the estimated mean values of the score test *T* ^*2*^ as a function of the sample size N, the number of causal SNPs, *M*, and whether the simulated normalized SNP variables *Z*_*j*_, *j* = 1…*M* are distributed with variance covariance matrix **I** or covariance matrix **K** with **K** being the model of moderate sample relatedness described above. In these simulations the true heritability of the trait *h* ^*2*^ was set to ½, therefore the heritability per SNP varies inversely with the number of markers. In order to compute power without relying too strictly on the asymptotic chi-square distribution of the test, we simulated its null behavior under the two different null hypotheses (i.e. *h* ^*2*^ = 0 with either **K** or **I** providing the alternative).

**Table 1:** Tabulations of the score test T^2^, empirical critical value and power to detect a 50% heritability over 2000 simulations for varying number of SNPs (M), numbers of observations (N) and relatedness matrix K.

N = 1000					
**Unrelated (K = I)**	**M = 120**	**M = 6,000**	**M = 50,000**	**M = 100,000**	**M = 300,000**
Mean	2,069.0	42.7	5.8	3.6	2.2
(SD)	(434.9)	(19.5)	(6.2)	(4.5)	(3.1)
Empirical Critical^+^ Value^+^	7.07	7.54	7.94	8.11	8.53
Empirical Power^++^	1	0.992	0.270	0.130	0.05
95% CI of Power	(1–1)	(0.990–0.994)	(0.257–0.283)	(0.120–0.140)	(0.044–0.056)
**Moderately related Off diag(K)~unif(0,.05)**					
Mean	1,765. 0	26.7	9.7	8.7	8.2
(SD)	(413.0)	(9.9)	(4.5)	(4.1)	(3.9)
Empirical Critical Value	6.57	4.85	4.27	4.50	4.29
Empirical Power	1	0.998	0.903	0.860	0.846
95% CI of Power	(1–1)	(0.997–1)	(0.894–0.912)	(0.850–0.870)	(0.834–0.857)
**N = 4000**					
**Unrelated (K = I)**	**M = 120**	**M = 6,000**	**M = 50,000**	**M = 100,000**	**M = 300,000**
Mean	33,080.0	666.8	80.7	41.0	15.1
(SD)	(5011.0)	(90.5)	(25.8)	(17.9)	(13.1)
Empirical Critical Value	7.74	7.44	7.88	7.47	7.72
Empirical Power	1	1	1	0.996	0.693
95% CI of Power	(1–1)	(1–1)	(1–1)	(0.994–0.998)	(0.679–0.706)
**Moderately related Off diag(K)~unif(0,.05)**					
Mean	31,520.0	441.5	146.9	131.2	121.7
(SD)	(5098.3)	(55.1)	(18.4)	(16.4)	(15.0)
Empirical Critical Value	6.89	4.81	4.00	3.96	3.88
Empirical Power	1	1	1	1	1
95% CI of Power	(1–1)	(1–1)	(1–1)	(1–1)	(1–1)

^+^ The empirical upper 5 percent critical value was computed under the null (h^2^ = 0) from 3,000 simulations

^++^ Power was computed as the fraction of simulations for which T^2^ was greater than the empirical critical value.

With a sample size of N = 1,000 and when normalized SNP variables **Z** have variance matrix **I** and number of markers, M, making up the heritability is either 120 or 6000, the simulated distribution of T^2^ is very far away from the empirically obtained test criteria and there is good power available to reject the null hypothesis that $\sigma_{g}^{2}=0$ and by implication also to estimate this quantity with reasonable accuracy. However for M = 50,000 or above the mean value of T^2^ falls markedly and very poor power for rejecting the null hypothesis is present with this sample size. When there is moderate relatedness between individuals in the study, for 50,000 markers or above the power of rejecting the null hypothesis that $\sigma_{g}^{2}>0$, is considerably larger than when all markers are independent; from M = 50,000–300,000 markers the power of detecting $\sigma_{g}^{2}>0$ is quite flat presumably indicating that the similarity matrix 1/M **ZZ’** has nearly converged to the true relationship matrix used in the simulation.

For the larger sample size of 4,000 there still is good power to reject the null hypothesis up to 100,000 markers irrespective of whether there is or is not mild population relatedness present. However when there is no hidden relatedness (**K** = **I**) there still remains a marked reduction in the average magnitude of the simulated *T* ^*2*^ as M increases and at 300,000 markers the estimated power falls to 69%.

We also notice some interesting behavior when examining the empirical type 1 error criteria in the various simulation conditions. For example with N = 1000 individuals under the null hypothesis of zero heritability and complete lack of relatedness the empirical criteria is larger (7.08) than the critical value (3.84) for a chi-square distribution with 1 degree of freedom even for M = 120 markers, and the empirical criteria gets larger as M increases. We interpret this as a failure of the score test to be completely reliable under the null because of a near lack of identifiability (due to the similarity between the matrix **I** and the matrix **G**), with **G** converging closer to **I** as the number of markers increase. With 4,000 individuals under the same conditions the empirical criteria are all elevated compared to the 3.84 nominal criteria, but no clear trend is evident as the number of markers increase. We expect that if many more markers were to be considered, that we would also see an increase in the empirical criteria, but we have not performed this experiment. For the moderately related individuals an opposite trend in the empirical type 1 error criteria with the number of markers is seen for both 1,000 individuals and 4,000 individuals. Now as the number of markers increases and hence **G** approaches the matrix **K** the empirical type 1 error criteria decreases and approaches the nominal criteria. We interpret this as due to better and better identifiability of the model as **G** becomes consistently closer to **K** than to **I**. Further details about the distribution of *T* ^*2*^ under the null hypothesis are given in S1 Table.

### Heritability Estimation Among Unrelated and Related Individuals

**Fig 1:**
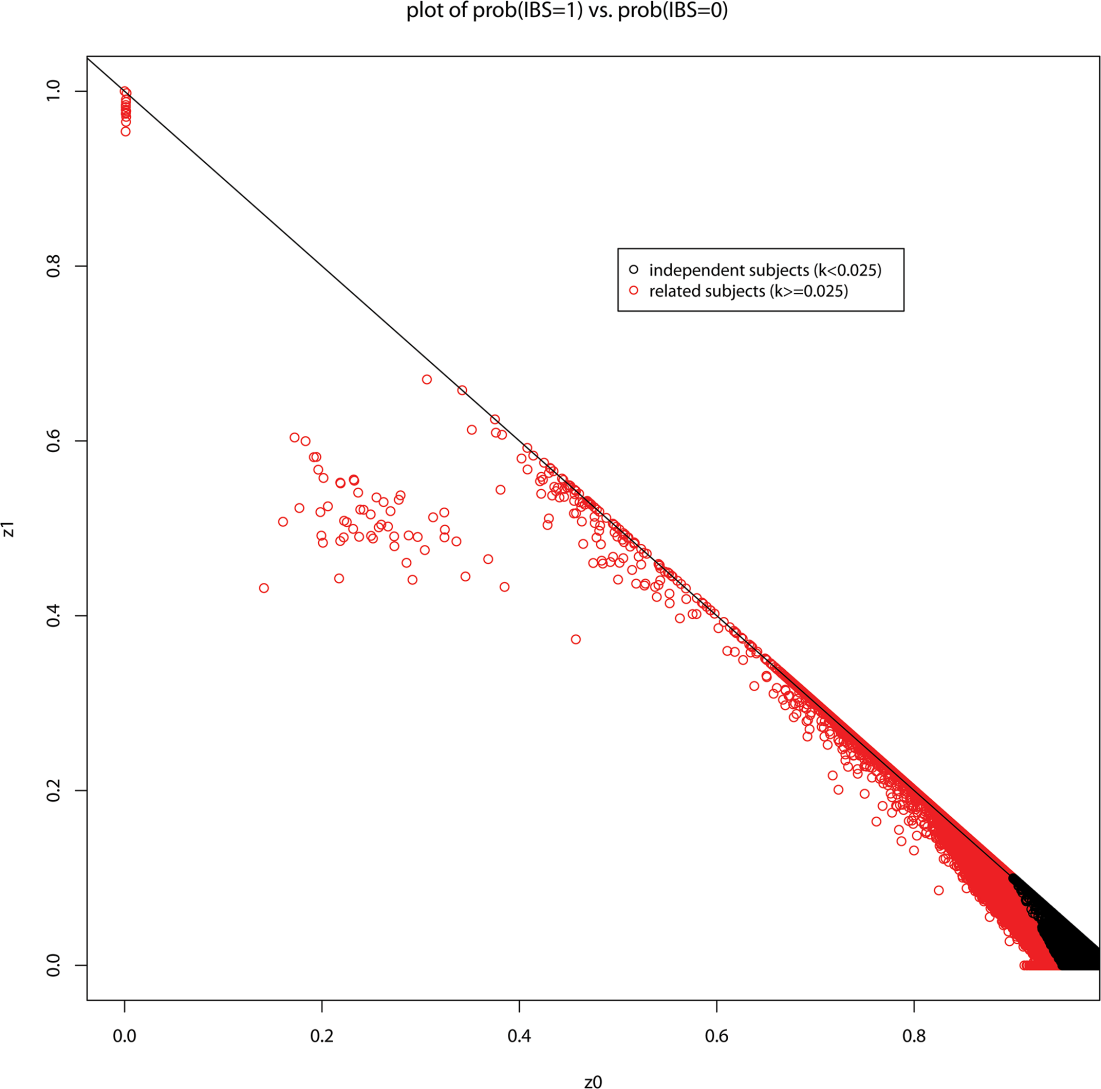
Relatedness in the African Ancestry Sample. A plot of the probability of sharing 1 allele identical by descent against probability of sharing zero alleles.

While we did not identify any monozygotic (MZ) twins or duplicate samples (z2 = 1), we found evidence of first-degree relatives (parent-offspring pairs and siblings) in the sample (Fig 1). Our independent sample (k<0.025) should have excluded possible parent-offspring pairs (z1 = 1), siblings or half siblings (z1 = 0.5) and cousins (z1 = 0.25). In the independent sample the 966,578 SNPs as a whole explain 44.7% (se: 3.7%, p<10^−16^) of the total variance of height, which is close to the fraction that Yang et al (2010) reported as being explained by 294, 831 common SNPs in a sample of Australian adolescents (44.9%; se: 8.4%; N = 3,925,p < 10^−7^) [3].

**Fig 2:**
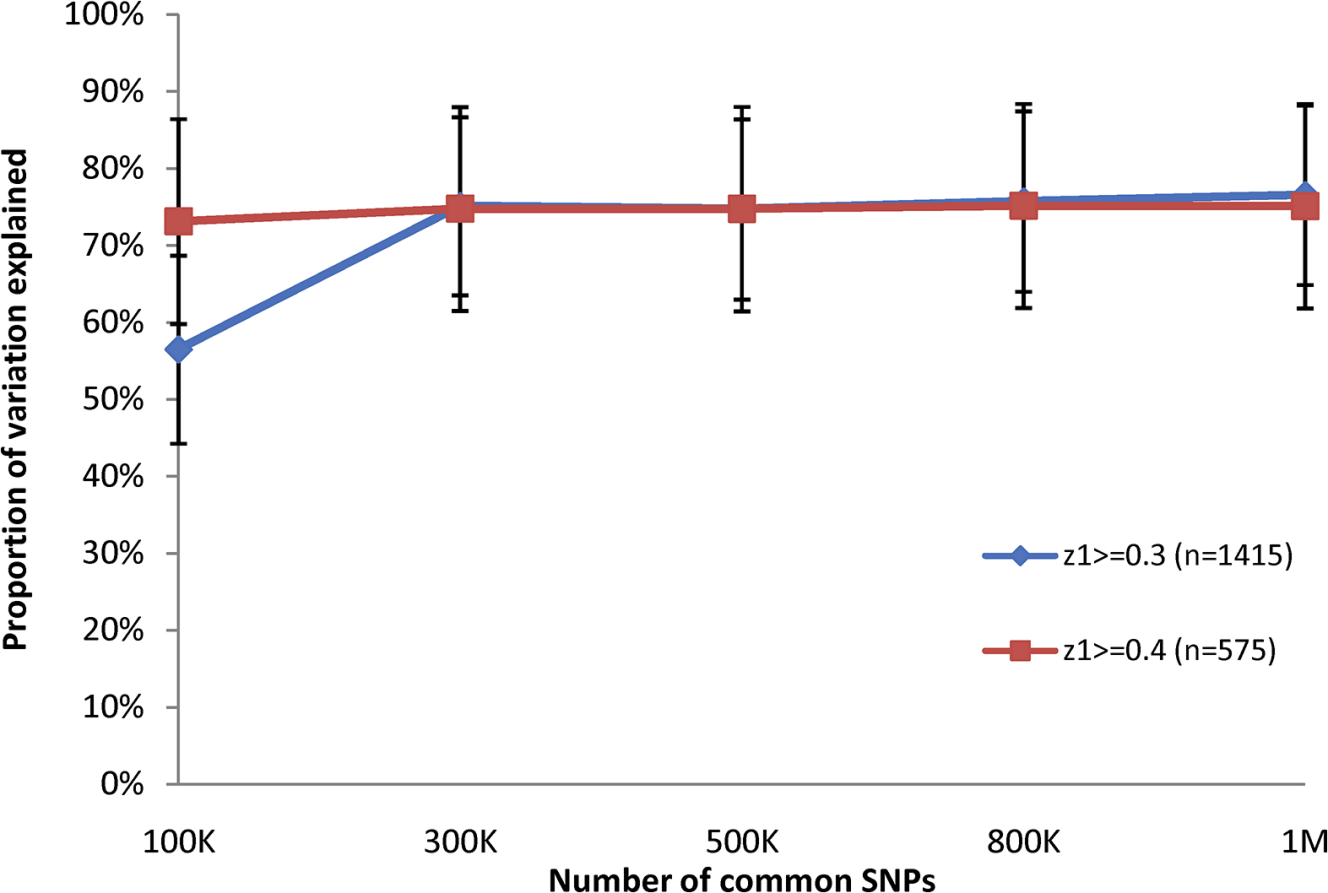
A comparison of phenotypic variation explained by different numbers of SNPs in close relatives. Variation of height explained by 10%, 30%, 50%, 80% and 100% of the nearly one million autosomal SNPs. Results are shown for two samples of related (z1> = 0.3, blue line and z1> = 0.4, red line) individuals.

A significant increase in the estimation of genetic variance component was noted, however, when considering closely related subjects. In the sample consisting of pairs of subjects with pair-wise z1> = 0.3, we found that 76.5% (se: 11.7%, p<10^−10^) of total phenotypic variance could be explained by the set of 966,578 SNPs, while in the sample where pair-wise z1> = 0.4, the fraction was 75.1% (se: 13.3%, p<10^−7^) (Fig 2). This is close to the 80% heritability of height estimated from twin studies [22–24]. Of course with highly related individuals it does not require 966,578 SNPs to estimate heritability. When we sampled 100,000 SNPs at random we estimated a heritability of 74.0%, which was statistically very consistent with the 75.1% estimated using all SNPs.

### Results of SNP Removal on Imputation *R* ^*2*^

Table 2 shows the distribution of the IMPUTE2 info score, an estimate of the squared correlation between imputed and true SNPs for the SNPs in the 1000 Genomes Study with minor allele frequency greater than 1% in the 1000 Genomes Study African ancestry samples.

**Table 2:** Effects of SNP removal on prediction of 1000 genomes SNPs using imputation based upon subsets of the 452,696 pruned SNPs.

1000 GenomesImputation	Min.	1st Qu.	Median	Mean	3rd Qu.	Max.
All SNPs	0	0.83	0.91	**0.87**	0.96	1.00
50% SNPs	0	0.70	0.80	**0.78**	0.86	1.00
25% SNPs	0	0.54	0.64	**0.64**	0.75	1.00
12% SNPs	0	0.49	0.48	**0.51**	0.57	1.00

Shown are the mean and quartiles of the distribution of estimated R^2^ values (IMPUTE2 info scores) for imputation of all variants found in the 1000 Genomes Study with frequency > 1% in African populations. The imputation basis consists of all (pruned) non-causal SNPs and randomly chosen subsets.

Table 3 gives the main results of the second simulation. The ability of the pruned SNPs, and subsets of the pruned SNPs, to predict the masked causal SNPs using the imputation methods described is characterized by the observed mean squared correlation between imputation dosage of causal SNPs and true genotypes in the main study panel. The mean value of observed *R* ^*2*^ (mean *R* ^*2*^ for imputation) was equal to 0.89 when all pruned SNPs were included, and steadily declined to 0.21 when only 12.5% of the pruned SNPs were used in the imputation basis. The 0.89 was higher than the target *R* ^*2*^ (.25 over a 50 SNP window) used in the PLINK pruning by a large margin, which appears to show that the haplotype-based methods used by IMPUTE2 are not greatly affected by the removal of nearby SNPs even when multivariate pruning is attempted. It is interesting to note when comparing Table 2 to Table 3, that the info scores for imputing the 1000 Genomes Study SNPs were considerably higher for the smaller SNP sets than the *R* ^*2*^ seen for the masked SNPs. While starting out very similarly, the drop in *R* ^*2*^ was much greater than the drop in info scores as SNPs were removed. This is likely a reflection of the pruning that we performed prior to picking the causal SNPs, i.e. our pruned SNPs were less predictable (on the basis of the observed SNPs) than typical SNPs in the 1000 Genomes Study. It is unclear however, why this is not also seen for the larger sets of SNPs (first two lines of Tables 2 and 3).

**Table 3:** Effects of SNP removal on prediction of masked causal SNPs using imputation based upon subsets of the 452,696 pruned SNPs.

Causal SNPs imputation	Min.	1st Qu.	Median	Mean	3rd Qu.	Max.
All non-causal SNPs	0.32	0.87	0.91	**0.89**	0.94	0.99
50% non-causal SNPs	0.11	0.70	0.80	**0.76**	0.86	0.97
25% non-causal SNPs	0.02	0.39	0.52	**0.51**	0.64	0.94
12% non-causal SNPs	0.00	0.11	0.19	**0.21**	0.29	0.89

Shown are the mean and quartiles of the distribution of estimated R^2^ values for imputation of the 1000 causal SNPs masked in the main study compared to actual unmasked genotypes. The imputation basis consists of all (pruned) non-causal SNPs and randomly chosen subsets.

### Results of SNP Removal on Heritability Estimation of Simulated Phenotypes

As expected when using the LMM on the simulated phenotypes we were able to recover most of the simulated heritability (set at 50%) using the actual genotypes for the unmasked causal SNPs (*h* ^*2*^ = 46.7%, se = 1.4%, first line of Table 4). However we noticed a marginally significant drop in the estimated heritability (to *h* ^*2*^ = 33.9%, se = 6.5%) when all 966,578 SNPs including the 1,000 causal SNPs were used simultaneously in the LMM, indicating that some of the signal from the causal SNPs was being swamped by the remaining SNPs. Since the causal SNPs were sampled randomly from the pruned SNPs we also ran the LMM using only the pruned (including causal) SNPs, the heritability was estimated to be only slightly higher (34.3% se = 7.8%) again indicating that the addition of non-causal SNPs seems to have partly swamped the LMM's dissection of signal from noise. When only the non-causal SNPs were used the estimated heritability dropped even further to 23.4% (se 7.3%) and further drops were seen as more SNPs were removed. The final estimate, using only 12.5% of the pruned SNPs was 6.8% (se = 4.3%).

**Table 4:** Effects of SNP removal on the estimated heritability of simulated phenotypes and actual self-reported height, using LMM.

	Simulated Phenotype		Observed Height	
SNP Set considered	*h* ^*2*^ (%)	se(*h* ^*2*^)	*h* ^*2*^(%)	se(*h* ^*2*^)
1000 causal SNPs	46.7	1.4		
All (Un-pruned) including Causal	33.9	6.5	53.2	6.8
All (Pruned) including Causal	34.3	7.8	62.3	8.3
All (Pruned) non-causal SNPs	23.4	7.3	62.6	8.3
50% non-causal SNPs	20.7	6.5	47.4	7.2
25% non-causal SNPs	13.2	5.4	33.6	5.9
12% non-causal SNPs	6.8	4.3	24.2	4.7

All analyses corrected for age, sex, study site, and 10 principal components. The un-pruned SNPs were those 966,578 passing quality control measures and the 452,696 pruned SNPs were selected by PLINK to reduce local LD. The 1000 causal SNPs were those used in the simulation of phenotype and were selected from among the pruned SNPs. The results are shown for the 6,244 individuals in the main study portion of the simulations for comparability to Table 3.

### Results of SNP Removal on Heritability Estimation of Self-reported Height

We next consider the results for the actual self-reported height data (last 2 columns of Table 4). As given above, using all SNPs and all 12,488 unrelated participants (i.e. ignoring the split into "reference panel" versus "main study") the heritability (as reported above) of the self-reported height data was estimated to be equal to 44.7%. Here however we focus (for the sake of comparability with Table 3) on the results from the 6,244 participants assigned to the main study for the purpose of the analysis. When heritability of height is estimated for these participants using all 966,578 SNPs a slightly higher *h* ^*2*^ is estimated 53.2% (se. 6.8%) than in all the participants but this clearly may be just a random fluctuation. Interestingly when only the pruned SNPs are used the heritability increased from 53.2% to 62.3% (se. 8.3%) Thereafter (as additional pruned SNPs were removed) *h* ^*2*^ decreased but not nearly as rapidly as for the simulated phenotype staying at 24.2% (se 4.7%) when only 12.5% (a total of 56,587 SNPs total) of the SNPs were used in the heritability analysis. We also noticed a similar pattern when all 12,488 individuals were included, with, for example, an increase to 52.3% (from 44.7%) observed with only the pruned SNPs.

## Discussion

Our first concern in this paper is whether the linear mixed model variance components approach to estimating heritability from GWAS data requires low levels of relatedness between individuals which would lead to a misattribution of overall additive heritability onto measured SNPs. Our simulations in Table 1 (of the score test in the presence or absence of hidden relatedness) indicate that this is in certain instances a well-founded concern since (1) even low levels of hidden relatedness can have an impact on the distribution of the score test and (2) that one cannot go on adding independent variables into the heritability analysis forever without seeing some loss of power. This is particularly the case for relatively small studies (N = 1,000 in Table 1) which appear to have very little power to estimate heritability using the LMM for large numbers of markers. However for larger studies of traits of strong heritability (as in the analysis of height presented here) this may not be an overriding issue.

Our analysis of the most closely related individuals in the study (Fig 2) provides further motivation of these concerns, as we find virtually the same estimate of additive heritability using 100,000 SNPs as for 996,578 SNPs. Clearly these SNPs simply need to estimate kinship coefficients between the highly related people and do not have to be in close LD with any phenotypically causal variants. Considering the 100,000 SNPs used as anything but delineators of overall relatedness would likely be a misattribution of the effects of unmeasured variants (and perhaps the effects of shared exposures, epistatic effects, etc.) onto the measured ones. We were concerned that the much lower levels of relatedness among individuals in GWAS studies may still be causing this kind of misattribution of effect from measured to unmeasured variants.

Our simulation experiment (simulated continuous phenotypes) did not however produce visible signs of misattribution of effect. In fact the heritability of the simulated phenotype, while well-captured (*h* ^*2*^ = 46.7% versus the simulated 50%) using only the 1000 simulated causal SNPs, was estimated to be considerably lower when either all the remaining SNPs (*h* ^*2*^ = 33.9%), or only the pruned SNPs (*h* ^*2*^ = 34.3%) were included in the model along with the 1000 causal SNPs (Table 4). When only the non-causal pruned SNPs were included heritability dropped by an additional factor of 31.8% (from 34.3% to 23.4%) which was much larger than the change in average imputation *R* ^*2*^ which went from 100% when the causal SNPs are included (i.e. the causal SNPs impute themselves perfectly) to 89% when only the non-causal pruned SNPs are used. Further relative drops in *h* ^*2*^ of 11.5% (23.4% to 20.7%), 36.4%, (20.7% to 13.2%) and 48.0% (13.2% to 0.68) respectively were seen as all but 50%, 25%, and 12% of SNPs were removed. These further relative drops in *h* ^*2*^ were only slightly less than the further relative drops in imputation *R* ^*2*^ (14.6%, 32.8%, 58.5%) as the same SNPs were removed. Comparing these later drops in *R* ^*2*^ versus *h* ^*2*^ do not imply strong evidence for misattribution in the sense that we see with close relatives (where the estimate of heritability is shown to be very similar over a wide range in SNP number).

Using the LMM, we estimated the heritability of human height in a sample of 14,419 subjects of African ancestry. We estimated that 44.7% of phenotypic variance of height was attributable to the additive effect of genetic variants in populations of African ancestry. In related samples, our results showed that the genetic component of variance explained about 75% of phenotypic variation. This estimate of *h* ^*2*^ would seem to imply that about 30% of height heritability may be due possibly to epistatic components or environmental sharing that should be less important among less closely related individuals than among close relatives or to the additive effect of other variants not in high LD with the markers, e.g. rare variants or common variants in poorly covered genetic regions.

When we focus on the results of our main experiment, some notable questions are raised about how to interpret the *h* ^*2*^ estimates from the LMM method. It appears to be naïve to take the estimate of *h* ^*2*^ coming from the method literally as the amount of variability that can be explained by the measured SNPs themselves. However, counter to our worries described above, in the simulation these estimates seem to be under-estimates rather than over-estimates of the portion of heritability that would be captured if all the causal SNPs are identified. In our simulation experiment the heritability estimate dropped from 46.7% (se 1.3%) to 34.3% (se 7.8%) simply by including the remaining pruned SNPs in the model and much further to 27.8% (se 7.3%) when the causal SNPs were removed even though the average *R* ^*2*^ for imputing the causal SNPs from the remainder of the pruned SNPs was equal to 0.89. This second drop is discrepant with the high *R* ^*2*^, it may have to do with the fact that in imputing causal SNPs the imputation methodology is making use of haplotype information rather than simple linear relationships between measured and unmeasured SNPs. Since the LMM method as implemented here and in many other papers is using a strictly linear kernel function, it may be missing the haplotype effects needed to impute the causal SNPs and hence to capture heritability. After this initial drop in *h* ^*2*^ further declines in *h* ^*2*^ and *R* ^*2*^ are somewhat more similar to each other comparing Table 3 to Table 4 although the significance of this is not very clear since they seem to be measuring different things.

For the actual self-reported height data the pattern of response of *h* ^*2*^ to SNP removal is strikingly different than for the simulated data. When only pruned SNPs are used estimated *h* ^*2*^ increases from 53.2% (in the main study using all SNPs) to 62.6% and then declines, but remains far higher than the analogous values of *h* ^*2*^ seen in the simulation. It is possible that the initial increase in heritability seen is explainable by the arguments of Speed et al [25] who found that increases in estimated *h* ^*2*^ resulted when regions which were rich in high LD SNPs were down weighted in the similarity matrix used for the LMM, which we can expect to be a result of pruning. However, this also was not reflected in our simulations; even though we selected causal SNPs from only pruned SNPs and not the full set of 966,578 SNPs, in the simulation heritability was only very slightly higher (34.3%, se 7.8%) when it was the pruned SNPs that were added to the causal SNPs than when it was the full set of SNPs added (33.9% se 6.5%), see Table 4.

The analysis of the actual self-reported height data is therefore quite far out of agreement with the simulation experiment, the drop off in heritability of the actual data is far slower than the drop off in heritability of the simulated data. One possible explanation for this is that the polygenic model that we simulated may not be polygenic enough, i.e. that there are far more than 1000 causal variants affecting height. If polygene contributions involve common causal variants lying nearly everywhere in the genome (each of course with very small effect), then we can expect that the G matrix for the causal variants would very much mimic the G matrix using all measured SNPs and even more so perhaps after pruning many SNPs out of regions with especially high LD (explaining the jump in *h* ^*2*^ after pruning).

Such an *infinitesimal polygene* [26] model implies that those contemplating functional work to better understand this phenotype, and by implication other phenotypes for which there is similar degree of heritability estimated in GWAS studies, may soon have a truly enormous number of candidates to sort through as large scale consortia continue on the path towards ever large sample sizes.

Putting aside this issue our simulations using the true genotypes available indicate that our GWAS-based estimates of heritability of height are not obviously biased upwards by subtle relatedness between the GWAS participants. Therefore concerning the central question, about whether today's GWAS data will need to be supplemented with additional variants in order to fully capture the additive heritability being estimated from LMM-based analyses, our results seem to support the idea that GWAS data collected today will have considerable future value as surrogates for as yet undiscovered causal variants, with the caveat that this value can be realized only if enough of causal variants can be identified and their effect sizes estimated. The concern about the necessity of a nearly infinitesimal model to explain our GWAS heritability results is that there may be so many causal variants and their effect sizes so small that characterizing enough of them to form a useful predictor of complex phenotypes may require unattainable levels of costs and resources.

## Supporting Information

S1 File Study Information. (DOCX)

S1 Table Tabulations of the score test T^2^. Empirical critical value under null hypothesis, and type I error given critical value 3.84 over 3000 simulations for varying number of SNPs (M), numbers of observations (N) and relatedness matrix **K**.
(DOCX)
