# Supplementary material for: Methodological Considerations in Estimation of Phenotype Heritability Using Genome-Wide SNP Data, Illustrated by an Analysis of the Heritability of Height in a Large Sample of African Ancestry Adults
Source: PLoS One. 2015 Jun 30;10(6):e0131106. doi: 10.1371/journal.pone.0131106 (PMC4488332; doi:10.1371/journal.pone.0131106)
Supplement: S1 File — (DOCX) [file pone.0131106.s001.docx]

This study includes African American women and men from 9 epidemiological studies of breast cancer and 14 epidemiological studies of prostate cancer, which comprise a total sample size of 15,032. Below is a brief description of each of the studies.

*The Multiethnic Cohort Study (MEC):* The MEC is a prospective cohort study of 215,000 men and women in Hawaii and Los Angeles [[1](#_ENREF_1)] between the ages of 45 and 75 years at baseline (1993-1996). Through December 31, 2007, a nested breast cancer case-control study in the MEC included 556 African American cases (544 invasive and 12 in situ) and 1,003 African American controls. An additional 178 African American breast cancer cases (ages: 50-84) diagnosed between June 1, 2006 and December 31, 2007 in Los Angeles County (but outside of the MEC) were included in the study. Through January 1, 2008 the African American case-control study in the MEC included 1,094 prostate cancer cases and 1,096 controls. An additional 746 prostate cancer cases from the MEC diagnosed after January 1, 2009 and 656 controls with GWAS data were included. All together, the MEC contributed 5,329 subjects to the height study.

*The Los Angeles component of The Women’s Contraceptive and Reproductive Experiences (CARE) Study:* The CARE Study is a large multi-center population-based case-control study that was designed to examine the effects of oral contraceptive (OC) use on invasive breast cancer risk among African American women and white women ages 35-64 years in five U.S. locations [[2](#_ENREF_2)]. Cases in Los Angeles County were diagnosed from July 1, 1994 through April 30, 1998, and controls were sampled by random-digit dialing (RDD) from the same population and time period; 380 African American cases and 224 African American controls were included in the study.

*The Women’s Circle of Health Study (WCHS):* The WCHS is an ongoing case-control study of breast cancer among European women and African American women in the New York City boroughs and in seven counties in New Jersey [[3](#_ENREF_3)]. Eligible cases included women with invasive breast cancer between 20 and 74 years of age; controls were identified through RDD. The WCHS contributed 272 invasive African American cases and 240 African American controls.

*The San Francisco Bay Area Breast Cancer Study (SFBCS):* The SFBCS is a population-based case-control study of invasive breast cancer in Hispanic, African American and non-Hispanic White women conducted between 1995 and 2003 in the San Francisco Bay Area [[4](#_ENREF_4)]. African American cases, ages 35-79 years, were diagnosed between April 1, 1995 and April 30, 1999, with controls identified through RDD. Included from this study were 172 invasive African American cases and 231 African American controls.

*The Northern California Breast Cancer Family Registry (NC-BCFR):* The NC-BCFR is a population-based family study conducted in the Greater San Francisco Bay Area, and one of 6 sites of the Breast Cancer Family Registry (BCFR) [[5](#_ENREF_5)]. African American breast cancer cases in NC-BCFR were diagnosed after January 1, 1995 and between the ages of 18 and 64 years; population controls were identified through RDD. Genotyping was conducted for 440 invasive African American cases and 53 African American controls.

*The Carolina Breast Cancer Study (CBCS):* The CBCS is a population-based case-control study conducted between 1993 and 2001 in 24 counties of central and eastern North Carolina [[6](#_ENREF_6)]. Cases were identified by rapid case ascertainment system in cooperation with the North Carolina Central Cancer Registry and controls were selected from the North Carolina Division of Motor Vehicle and United States Health Care Financing Administration beneficiary lists. Participants’ ages ranged from 20 to 74 years. DNA samples were provided from 656 African American cases with invasive breast cancer and 608 African American controls.

*The Prostate, Lung, Colorectal, and Ovarian Cancer Screening Trial (PLCO) Cohort*: The Prostate, Lung, Colorectal, and Ovarian Cancer Screening Trial [[7](#_ENREF_7)], is a randomized, two-arm trial among men and women aged 55-74 years to determine if screening reduced the mortality from these cancers. Male participants randomized to the intervention arm underwent prostate specific antigen (PSA) screening at baseline and annually for 5 years and digital rectal examination at baseline and annually for 3 years. Sequential blood samples were collected from participants assigned to the screening arm; participation was 93% at the baseline blood draw (1993-2001). Buccal cell samples were collected from participants in the control arm of the trial; participation was about 85% for this component. A total of 64 African American invasive breast cancer cases and 133 African American controls, as well as 286 African American prostate cancer cases and 269 controls without a history of prostate cancer contributed to this study.

*The Nashville Breast Health Study (NBHS):* The NBHS is a population-based case-control study of incident breast cancer conducted in Tennessee [[8](#_ENREF_8)]. The study was initiated in 2001 to recruit patients with invasive breast cancer or ductal carcinoma in situ, and controls, recruited through RDD between the ages of 25 and 75 years. NBHS contributed 310 African American cases (57 in situ), and 186 African American controls.

*Wake Forest University Breast Cancer Study (WFBC):* African American breast cancer cases and controls in WFBC were recruited at Wake Forest University Health Sciences from November 1998 through December 2008 [[9](#_ENREF_9)]. Controls were recruited from the patient population receiving routine mammography at the Breast Screening and Diagnostic Center. Age range of participants was 30-86 years. WFBC contributed 125 cases (116 invasive and 9 in situ) and 153 controls to the analysis.

*The Southern Community Cohort Study (SCCS):* The SCCS is a prospective cohort of African and non-African Americans which during 2002-2009 enrolled approximately 86,000 residents aged 40-79 years across 12 southern states [[10](#_ENREF_10)].  Recruitment occurred mainly at community health centers, institutions providing basic health services primarily to the medically uninsured, so that the cohort includes many adults of lower income and educational status. Each study participant completed a detailed baseline questionnaire, and nearly 90% provided a biologic specimen (approximately 45% a blood sample and 45% buccal cells). Follow-up of the cohort is conducted by linkage to national mortality registers and to state cancer registries. Included in this study are 212 incident African American prostate cancer cases and a matched stratified random sample of 419 African American male cohort members without prostate cancer at the index date selected by incidence density sampling. We included an additional 51 incident and prevalent cases from the SCCS (incident cases diagnosed after June 1, 2006) and 104 controls with GWAS data.

*The Cancer Prevention Study II Nutrition Cohort (CPS-II).*The CPS-II Nutrition Cohort includes over 86,000 men and 97,000 women from 21 US states who completed a mailed questionnaire in 1992/93 (aged 40-94 years at baseline) [[11](#_ENREF_11)]. Starting in 1997, follow-up questionnaires were sent to surviving cohort members every other year to update exposure information and to ascertain occurrence of new cases of cancer; a >85% response rate has been achieved for each follow-up questionnaire. From 1998-2001, blood samples were collected in a subgroup of 39,380 cohort members. To further supplement the DNA resources, during 2001-2002, buccal cell samples were collected by mail from an additional 70,004 cohort members. Incident cancers are verified through medical records, or through state cancer registries or death certificates when the medical record cannot be obtained. Genomic DNA from 76 African American prostate cancer cases and 152 age-matched controls were included in stage 1 of the scan.

*Prostate Cancer Case-Control Studies at MD Anderson (MDA):* Participants in this study were identified from epidemiological prostate cancer studies conducted at the University of Texas M.D. Anderson Cancer Center in the Houston Metropolitan area since 1996 Cases were accrued from six institutions in the Houston Medical Center and were no restricted with respect to Gleason score, stage or PSA. Controls were identified via random-digit-dialing or among hospital visitors and they were frequency matched to cases on age and race. Lifestyle, demographic, and family history data were collected using a standardized questionnaire. These studies contributed 543 African American cases and 474 controls to this study [[12](#_ENREF_12)].

*Identifying Prostate Cancer Genes (IPCG):* Cases in this study were patients 1) undergoing treatment for prostate cancer in the Department of Urology at Johns Hopkins Hospital from 1999 to 2007; 2) undergoing treatment at the Sidney Kimmel Comprehensive Cancer Center from 2003 to 2007; and 3) outside referrals as part of the Hereditary Prostate Cancer Study from 1990 to present. Blood was obtained from groups 2) and 3) while DNA from normal tissue was obtained from group 1). Data are available on age at diagnosis, race, pretreatment prostate-specific antigen (PSA) values, clinical pathology values, and family history. The control subjects were men undergoing disease screening and were not thought to have prostate cancer on the basis of a physical exam and a serum PSA value below 4ng/ml. Screenings were performed at the Johns Hopkins Applied Physics Lab, at Bethlehem Steel in Baltimore, and at local African American churches in East Baltimore[[13](#_ENREF_13)]. A total of 368 African American cases and 172 controls contributed to this consortium.

*The Los Angeles Study of Aggressive Prostate Cancer (LAAPC):* The LAAPC is a population-based case-control study of aggressive prostate among African Americans in Los Angeles County [[14](#_ENREF_14)]. Cases were identified through the Los Angeles County Cancer Surveillance Program rapid case ascertainment system and eligible cases included African American men diagnosed with a first primary prostate cancer between January 1, 1999 and December 31, 2003. Eligible cases also had either tumor extension outside the prostate, metastatic prostate cancer in sites other than prostate, or needle biopsy of the prostate with Gleason grade 8 or higher, or Gleason grade 7 and tumor in more than 2/3 of the biopsy cores. Controls were identified by a neighborhood walk algorithm and were men never diagnosed with prostate cancer, and were frequency matched to cases on age (±5 years). For this study, genomic DNA was included for 296 cases and 140 controls. We also included an additional 163 African American controls from the MEC that were frequency matched to cases on age.

*Prostate Cancer Genetics Study (CaP Genes):* The African-American component of this study population comprised 160 men: 75 cases diagnosed with more aggressive prostate cancer and 85 age-matched controls [[14](#_ENREF_14)]. All subjects were recruited and frequency-matched on the major medical institutions in Cleveland, Ohio (i.e., the Cleveland Clinic, University Hospitals of Cleveland, and their affiliates) between 2001 and 2004. The cases were newly diagnosed with histologically confirmed disease: Gleason score 7; tumor stage T2c; or a prostate-specific antigen level >10 ng/ml at diagnosis. Controls were men without a prostate cancer diagnosis who underwent standard annual medical examinations at the collaborating medical institutions.

*Case-Control Study of Prostate Cancer among African Americans in Washington, DC (DCPC):* Unrelated men self-described as African American were recruited for several case-control studies on genetic risk factors for prostate cancer between the years 2001 and 2005 from the Division of Urology at Howard University Hospital (HUH) in Washington, DC. Control subjects unrelated to the cases and matched for age (± 5 years) were also ascertained from the prostate cancer screening population of the Division of Urology at HUH [[15](#_ENREF_15)]. These studies included 292 cases and 359 controls.

*King County (Washington) Prostate Cancer Studies (KCPCS):* The study population consists of participants from one of two population-based case-control studies among residents of King County, Washington [[16](#_ENREF_16),[17](#_ENREF_17)]. Incident Caucasian and African American cases with histologically confirmed prostate cancer were ascertained from the Seattle-Puget Sound SEER cancer registry during two time periods, 1993-1996 and 2002-2005.Age-matched (5-year age groups) controls were men without a self-reported history of being diagnosed with prostate cancer and were identified using one-step random digit telephone dialing. Controls were ascertained during the same time periods as the cases. A total of 145 incident African American cases and 81 African American controls were included from these studies.

*The Gene-Environment Interaction in Prostate Cancer Study (GECAP):* The Henry Ford Health System (HFHS) recruited cases diagnosed with adenocarcinoma of the prostate of Caucasian or African-American race, less than 75 years of age, and living in the metropolitan Detroit tri-county area [[18](#_ENREF_18)]. Controls were randomly selected from the same HFHS population base from which cases were drawn. The control sample was frequency matched at a ratio of 3 enrolled cases to 1 control based on race and five-year age stratum. In total, 637 cases and 244 controls were enrolled between January 2002 and December 2004.  Of study enrollees, DNA for 234 African Americans cases and 92 controls were included in stage 1 of the scan.

*Prostate Cancer in a Black Population (PCBP):* The PCBP is a population-based case-control study of prostate cancer conducted in Barbados, West Indies [[19](#_ENREF_19)]. The study (2002-2011) included all incident, histologically-confirmed cases of prostate cancer ascertained from the Pathology Department of the Queen Elizabeth Hospital, Bridgetown, the only institution on the island where specimens are evaluated. Controls were randomly selected from a national database and frequency matched (by 5-year age groups) to the cases. We included 238 prostate cancer cases and 231 controls with GWAS data.

*North Carolina Prostate Cancer Study (NCPCS):*NCPCS is a population-based case-control study in the Western part of North Carolina (NC)[[20](#_ENREF_20)]. This study population included 216 cases and 249 controls that were recruited from November 2006 to November 2008.  Cases were identified via the Rapid Case Ascertainment (RCA) center of the North Carolina Central Cancer Registry (NCCCR), which collects standardized demographic and clinical data on every case of cancer diagnosed in NC, as mandated by state law. Inclusion criteria for cases are a new histological diagnosis of prostate cancer as documented by the NCCCR, age 40 to 70 years, and residence within 12 contiguous NC counties.  Case exclusion criteria were prostate cancer incorrectly reported to the NCCCR (false reports), residence in a rest home, hospital, or hospice, any health condition that does not allow completion of the interview, and inability to obtain contact information. Controls were recruited via a friend referral method, and their inclusion criteria required a match to the age, race, and residence county of a case. Control exclusion criteria were a previous diagnosis of prostate cancer, residence in a rest home, hospital, or hospice, and a health condition that does not allow completion of the interview.  All cases and controls completed the same participation process, consisting of a blood sample (for DNA and serum), Food Frequency Questionnaire (NIH), and a medical/family history questionnaire.

*Selenium and Vitamin E Cancer Prevention Trial (SELECT):* SELECT is a phase III, placebo-controlled trial that tested whether selenium and vitamin E might reduce the risk of developing prostate cancer [[21](#_ENREF_21)]. A total of 35,534 men 55 and older (50 years and older for African Americans) without a history of prostate cancer were enrolled between 2001 and 2004. About 12% of the SELECT participants are African American. A case-cohort study has been established in SELECT and, as of December 31, 2009, includes 217 African American prostate cancer cases and 222 African American non-cases, all of which are included in the analysis.

**References**

1. Kolonel LN, Henderson BE, Hankin JH, Nomura AM, Wilkens LR, et al. (2000) A multiethnic cohort in Hawaii and Los Angeles: baseline characteristics. Am J Epidemiol 151: 346-357.

2. Marchbanks PA, McDonald JA, Wilson HG, Burnett NM, Daling JR, et al. (2002) The NICHD Women's Contraceptive and Reproductive Experiences Study: methods and operational results. Ann Epidemiol 12: 213-221.

3. Ambrosone CB, Ciupak GL, Bandera EV, Jandorf L, Bovbjerg DH, et al. (2009) Conducting Molecular Epidemiological Research in the Age of HIPAA: A Multi-Institutional Case-Control Study of Breast Cancer in African-American and European-American Women. J Oncol 2009: 871250.

4. John EM, Schwartz GG, Koo J, Wang W, Ingles SA (2007) Sun exposure, vitamin D receptor gene polymorphisms, and breast cancer risk in a multiethnic population. Am J Epidemiol 166: 1409-1419.

5. John EM, Hopper JL, Beck JC, Knight JA, Neuhausen SL, et al. (2004) The Breast Cancer Family Registry: an infrastructure for cooperative multinational, interdisciplinary and translational studies of the genetic epidemiology of breast cancer. Breast Cancer Res 6: R375-389.

6. Newman B, Moorman PG, Millikan R, Qaqish BF, Geradts J, et al. (1995) The Carolina Breast Cancer Study: integrating population-based epidemiology and molecular biology. Breast Cancer Res Treat 35: 51-60.

7. Gohagan JK, Prorok PC, Hayes RB, Kramer BS (2000) The Prostate, Lung, Colorectal and Ovarian (PLCO) Cancer Screening Trial of the National Cancer Institute: history, organization, and status. Control Clin Trials 21: 251S-272S.

8. Zheng W, Cai Q, Signorello LB, Long J, Hargreaves MK, et al. (2009) Evaluation of 11 breast cancer susceptibility loci in African-American women. Cancer Epidemiol Biomarkers Prev 18: 2761-2764.

9. Smith TR, Levine EA, Freimanis RI, Akman SA, Allen GO, et al. (2008) Polygenic model of DNA repair genetic polymorphisms in human breast cancer risk. Carcinogenesis 29: 2132-2138.

10. Signorello LB, Hargreaves MK, Steinwandel MD, Zheng W, Cai Q, et al. (2005) Southern community cohort study: establishing a cohort to investigate health disparities. J Natl Med Assoc 97: 972-979.

11. Calle EE, Rodriguez C, Jacobs EJ, Almon ML, Chao A, et al. (2002) The American Cancer Society Cancer Prevention Study II Nutrition Cohort: rationale, study design, and baseline characteristics. Cancer 94: 2490-2501.

12. Strom SS, Gu Y, Zhang H, Troncoso P, Babaian RJ, et al. (2004) Androgen receptor polymorphisms and risk of biochemical failure among prostatectomy patients. Prostate 60: 343-351.

13. Gudmundsson J, Sulem P, Manolescu A, Amundadottir LT, Gudbjartsson D, et al. (2007) Genome-wide association study identifies a second prostate cancer susceptibility variant at 8q24. Nat Genet 39: 631-637.

14. Ingles SA, Coetzee GA, Ross RK, Henderson BE, Kolonel LN, et al. (1998) Association of prostate cancer with vitamin D receptor haplotypes in African-Americans. Cancer Res 58: 1620-1623.

15. Robbins C, Torres JB, Hooker S, Bonilla C, Hernandez W, et al. (2007) Confirmation study of prostate cancer risk variants at 8q24 in African Americans identifies a novel risk locus. Genome Res 17: 1717-1722.

16. Agalliu I, Salinas CA, Hansten PD, Ostrander EA, Stanford JL (2008) Statin use and risk of prostate cancer: results from a population-based epidemiologic study. Am J Epidemiol 168: 250-260.

17. Stanford JL, Wicklund KG, McKnight B, Daling JR, Brawer MK (1999) Vasectomy and risk of prostate cancer. Cancer Epidemiol Biomarkers Prev 8: 881-886.

18. Rybicki BA, Neslund-Dudas C, Nock NL, Schultz LR, Eklund L, et al. (2006) Prostate cancer risk from occupational exposure to polycyclic aromatic hydrocarbons interacting with the GSTP1 Ile105Val polymorphism. Cancer Detect Prev 30: 412-422.

19. Nemesure B, Wu SY, Hennis A, Leske MC, Prostate Cancer in a Black Population Study G (2012) Central adiposity and Prostate Cancer in a Black Population. Cancer Epidemiol Biomarkers Prev 21: 851-858.

20. Xu J, Kibel AS, Hu JJ, Turner AR, Pruett K, et al. (2009) Prostate cancer risk associated loci in African Americans. Cancer Epidemiol Biomarkers Prev 18: 2145-2149.

21. Lippman SM, Goodman PJ, Klein EA, Parnes HL, Thompson IM, Jr., et al. (2005) Designing the Selenium and Vitamin E Cancer Prevention Trial (SELECT). J Natl Cancer Inst 97: 94-102.
