# Supplementary material for: Methodological Considerations in Estimation of Phenotype Heritability Using Genome-Wide SNP Data, Illustrated by an Analysis of the Heritability of Height in a Large Sample of African Ancestry Adults
Source: PLoS One. 2015 Jun 30;10(6):e0131106. doi: 10.1371/journal.pone.0131106 (PMC4488332; doi:10.1371/journal.pone.0131106)
Supplement: S1 Table — Empirical critical value under null hypothesis, and type I error given critical value 3.84 over 3000 simulations for varying number of SNPs (M), numbers of observations (N) and relatedness matrix K. (DOCX) [file pone.0131106.s002.docx]

S1 Table. Tabulations of the score test T^2^. Empirical critical value under null hypothesis, and type I error given critical value 3.84 over 3000 simulations for varying number of SNPs (M), numbers of observations (N) and relatedness matrix **K**.

| **N=1000** | | | | | |
| --- | --- | --- | --- | --- | --- |
| **Unrelated (K=I)** | **M=120** | **M=6,000** | **M=50,000** | **M=100,000** | **M=300,000** |
| Mean | 1.89 | 2.02 | 2.14 | 2.06 | 2.26 |
| (SD) | (2.7) | (2.8) | (2.9) | (2.9) | (3.15) |
| Empirical Critical Value^+^ | 7.08 | 7.54 | 7.94 | 8.11 | 8.54 |
| Type I Error ^++^ | 0.16 | 0.17 | 0.18 | 0.17 | 0.19 |
|  |  |  |  |  |  |
| **Moderately related Off diag(K)~unif(0,.05)** | | | | | |
| Mean | 1.75 | 1.44 | 1.32 | 1.36 | 1.33 |
| (SD) | (2.4) | (1.7) | (1.5) | (1.6) | (1.5) |
| Empirical Critical Value | 6.57 | 4.85 | 4.27 | 4.50 | 4.29 |
| Type I Error | 0.14 | 0.10 | 0.07 | 0.08 | 0.07 |
|  |  |  |  |  |  |
| **N=4000** | | | | | |
| **Unrelated (K=I)** | **M=120** | **M=6,000** | **M=50,000** | **M=100,000** | **M=300,000** |
| Mean | 2.02 | 1.99 | 2.04 | 1.94 | 2.09 |
| (SD) | (2.9) | (2.8) | (2.8) | (2.7) | (2.9) |
| Empirical Critical Value | 7.74 | 7.44 | 7.88 | 7.47 | 7.72 |
| Type I Error | 0.17 | 0.17 | 0.17 | 0.16 | 0.17 |
|  |  |  |  |  |  |
| **Moderately related Off diag(K)~unif(0,.05)** | | | | | |
| Mean | 1.83 | 1.40 | 1.25 | 1.25 | 1.26 |
| (SD) | (2.5) | (1.7) | (1.4) | (1.4) | (1.3) |
| Empirical Critical Value | 6.89 | 4.81 | 4.00 | 3.96 | 3.88 |
| Type I Error | 0.15 | 0.09 | 0.06 | 0.06 | 0.05 |

^+^ The empirical critical value is calculated as the 95% percentile cutoff of all replicates for each scenario.

^++^ The type I error is calculated as the fraction of replicates with score statistics larger than 3.84.
